# Supplementary material for: WordCommentsAnalyzer: A windows software tool for qualitative research
Source: F1000Res. 2018 Sep 4;7:536. Originally published 2018 May 3. [Version 2] doi: 10.12688/f1000research.14819.2 (PMC6137411; doi:10.12688/f1000research.14819.2)
Supplement: Supplementary file 3 [file f1000research-7-17614-s0002.tgz › b2a79496-4ada-47c4-9682-a99ce867836d.zip!/Eight Iranian Health Journals/Journal of Research in Rehabilitation Sciences _53.docx]

© Journal of Research in Rehabilitation Sciences . This work is licensed under CC BY-NC 4.0 (https://creativecommons.org/licenses/by-nc/4.0/).

Volume & Issue: 1386

Title: هم ـ فعالیتی عضلات در مدلهای بیومکانیکی

http://jrrs.mui.ac.ir/index.php/jrrs/article/view/93

چکیده مقدمه: هم ‌ـ ‌فعالیتی‌ (co-activation)‌عضلات‌ مفهومی ‌پر ‌اهمیت ‌در ‌مطالعات بیومکانیکی و توانبخشی مفاصل ستون فقرات و زانو است که به عنوان معیاری در طرح ريزي استراتژيهاي توانبخشي و ارزیابی اقدامات کلینیکی، به وفور مورد استفاده محققين توانبخشي و همكاران باليني قرار می‌گیرد. در اين مقاله بطور خلاصه علل ظهور تبعات بيومكانيكي و فيزيولوژيك هم‌ ـ فعالیتي عضلات، با استفاده از چند مثال توضیح داده می شود. اميد است اين نوشتار لزوم همكاري و تعامل بيشتر گروه‌هاي باليني‌و‌‌‌‌مهندسي‌بيومكانيك‌برای توسعه مدلهاي بيومكانيكي، همیاری در طرح‌ریزی تحقيقات تجربي و روشهاي دقیق‌تر ارزیابی عملکرد عصبی ـ عضلانی در فعالیتهای فانکشنال را بیشتر بیان کند.

Title: بررسي تكرارپذيري تستهاي باليني استاتيك،‌ ديناميك و برانگيزنده درد مفصل ساكروايلياك

http://jrrs.mui.ac.ir/index.php/jrrs/article/view/94

چکیده مقدمه: در مورد قابليت تكرارپذيري بسياري از تستهاي استاتيك، ديناميك و برانگيزنده درد كه به طور رايج براي تشخيص ديسفانكشن هاي مفصل ساكروايلياك به كار مي‌روند اختلاف نظر وجود دارد. مواد و روش‌ها:  تست‌هاي استاتيك مورد بررسي در اين تحقيق لمس و ارزيابي‌ زوائد Posterior Superior Iliac Spine (PSIS) در حالت ايستاده و نشسته، لمس و ارزيابي زوائد Anterior Superior Iliac Spine (ASIS) در حالت ايستاده، لمس و ارزيابي قوزكهاي داخلي در حالت طاقباز و نشسته بودند. تستهاي ديناميك مورد مطالعه Gillet، Standing flexion و Sitting flexion  بودند. اين تست ها توسط چهار آزمونگر بر روي ده دختر سالم انجام شدند. هر كدام از اين تستها چهار بار و در طي سه روز توسط هر آزمونگر انجام شدند كه در جمع 1280 ارزيابي انجام گرفت. تستهاي برانگيزنده درد  شامل تست برانگيزنده درد خلف لگن (Posterior Pelvic Pain Provocation test - PPPPT) ، تست پاتريك و ابداكسيون مقاومتي هيپ بودند. اين تست ها توسط دو آزمونگر بر روي 20 بيمار مبتلا به كمردرد مزمن انجام گرفتند. هر كدام از اين تستها دو بار توسط هر آزمونگر بر روي نمونه هاي مورد بررسي انجام شدند. نتايج: ميانگين مقادير كاپا براي تكرارپذيري داخل آزمونگر ارزيابي زوائد PSIS در حالت ايستاده و نشسته به ترتيب 48/0 و 24/0، ارزيابي زوائد ASIS 24/0، ارزيابي قوزكهاي داخلي در حالت طاقباز و نشسته به ترتيب 25/0 و 21/0 بود. مقادير آزمون كاپا براي تست هاي Gillet، Standing flexion و Sitting flexion به ترتيب 12/0، 19/0 و 13/0 بود. تكرارپذيري بين آزمونگر تمام تستهاي استاتيك و ديناميك در حد خفيف (19/0- 12/0) بدست آمد. با استفاده از آزمون كاپا تكرارپذيري بين آزمونگر تستهاي PPPPT، پاتريك و ابداكسيون مقاومتي هيپ براي پاي راست به ترتيب 7/0، 7/0 و 6/0 و براي پاي چپ 7/0، 78/0  34/0 بدست آمد. ميانگين تكرارپذيري داخل آزمونگر تستهاي PPPPT، پاتريك و ابداكسيون مقاومتي هيپ بين 75/0 و 91/0  بدست آمد.  بحث: بر اساس نتايج اين تحقيق قابليت تكرارپذيري تستهاي استاتيك و ديناميك مفصل ساكروايلياك مورد ترديد بوده استفاده باليني از آنها براي تشخيص ديسفانكشن هاي اين مفصل بايد با احتياط همراه باشد. همچنين در مورد تستهاي برانگيزنده درد پيشنهاد مي شود كه از تستهاي PPPPT و ابداكسيون مقاومتي هيپ به عنوان تستهائي با تكرارپذيري بالا براي تشخيص پاتولوژيهاي مفصل ساكروايلياك استفاده كرد.

Title: بررسی و مقایسه مهارتهای ادراک بینایی درکودکان نارساخوانی رشدی و عادی شهر اهواز

http://jrrs.mui.ac.ir/index.php/jrrs/article/view/95

چکیده مقدمه: یکی از شگفت انگیزترین مهارتهای انسانی، خواندن می­باشد. ما که از کودکی با خواندن آشنا بوده­ایم و راحت و روان از عهدۀ آن برمی‌آییم، کمتر به پیچیدگی مکانیسم این مهارت توجه می‌کنیم. نارساخوانی رشدی یکی از انواع اختلالات خواندن است. محققین مختلف نارساخوانی رشدی را از دیدگاههای مختلف مورد بررسی قرار داده‌اند و هر یک سعی در روشن کردن علل بوجود آورنده و مکانیسم‌های زیربنایی ایجاد نارساخوانی داشته­اند. یکی از فرضیات مطرح در زمینه علت­شناسی این اختلال وجود نقایص ادراک بینایی در این کودکان است. هدف از پژوهش حاضر بررسی مهارتهای ادراک بینایی نارساخوانهای رشدی و مقایسه این مهارت­ها با کودکان عادی است. مواد و روش‌ها: این پژوهش از نوع مقطعی و تحلیلی است. جامعه مورد مطالعه 35 کودک نارساخوان رشدی و 35 کودک عادی از مدارس ابتدایی مناطق 1، 2، 3 و 4 آموزش و پرورش شهر اهواز می‌باشند. مهارتهای ادراک بینایی با استفاده از آزمون مهارتهای ادراک بینایی TVPS-R   بررسی شده­اند. نتايج: نتایج این پژوهش نشان می­دهند که مهارتهای ادراک بینایی کودکان نارساخوان در مقایسه با کودکان عادی تفاوت معني­داری ندارند. این یافته­ها با نظریاتی همسو می­باشند که علت نارساخوانی رشدی را در نقایص بینایی به تنهایی جستجو نمی­کنند و علتهای دیگری را برای توجیه این اختلال ضروری می­دانند.

Title: اثرات ليزر در نقاط طب سوزني بر روي سردردها ي تنشي مزمن

http://jrrs.mui.ac.ir/index.php/jrrs/article/view/96

چکیده مقدمه: سردرد بر كيفيت زندگي بسياري از مردم در سرتاسر جهان اثر مي­گذارد . سردرد تنشي از جمله رايج ترين آنهاست. طب سوزني گسترده ترين روش درماني معمول براي سردرد ها مي‌باشد. هدف اين مطالعه بررسي آثار طب سوزني ليزري در اينگونه سردردهاست. مواد و روش­ها:  پنجاه بيمار داراي سردرد نوع تنشي ـ مزمن بصورت تصادفي به دو گروه درمان و دارو نما دسته بندي گرديدند. بيماران گروه درمان طب سوزني ليزري كم انرژي در نقاط 7LU و 4LI و 20GB و 14GB بصورت دو طرفه (دوجانبه ) دريافت نمودند. نقاط مزبور به مدت 43 ثانيه در معرض تابشهاي با شدت J3/1 (تقربياً  J/ cm213) قرار گرفتند جلسات درماني به صورت 3 جلسه در هفته وكلا 10 جلسه بود. گروه دارونما نيز به همين سبك در معرض قرار گرفتند  به جز اين كه توان خروجي دستگاه صفر بود. متغيرهاي مورد مطالعه عبارت بودند از شدت سردرد Visual Analogue Scale) (VAS)) طول مدت حمله ها  و تعداد روزهايي كه در طول ماه سردرد داشتند كه طي يادداشت هاي روزانه بصورت ماهانه و تا سه ماه بعد از درمان مورد ارزيابي قرار مي گرفت. نتايج: تفاوتهاي قابل ملاحظه­ای بين گروهها (001/0 > p ) در تغيير از، خط پايه در ماههاي اول، دوم و سوم در ميانگين نمره شدت سردرد (گروه درماني5-، 3-، 2-  وگروه دارونما 1-، 0 و0 ) در ميانگين مدت زمان حمله ها (گروه درماني 6- ، 4 - و 4 - ساعت وگروه دارونما 1-، 0 و 0 ساعت )، و در میانگین تعداد  روزهاي با سردرد در هر ماه (گروه درماني 15- و10- و 8 - وگروه دارونماي 2-، 0 و 0) وجود داشت. بحث: اين بررسي مبين آن است كه طب سوزني ليزري مي تواند يك درمان مؤثر براي سردرد نوع تنشي مزمن باشد، اما نتايج حاصله بايد در آزمايشهاي وسيع­تر و دقيق­تر تأييد گردند.

Title: بررسي مقايسه اي خصوصيات دو نوع پنجه SACH FOOT ايراني و آلماني با استفاده از دستگاه آناليز گيت و دينامومتر

http://jrrs.mui.ac.ir/index.php/jrrs/article/view/97

چکیده مقدمه: با توجه به شيوع قطع عضو اندام تحتاني و درصد بالاي قطع عضو زير زانو، پنجه بكار گرفته شده در پروتز هاي افراد نيازمند از اهميت خاصي برخوردار است. بيشترين كاربرد پنجه در پروتزهاي زير زانو از نوع SACH Foot  مي‌باشد. شركتها و مراكز تحقيقاتي زيادي دست اندركار ساخت و طراحي اين نوع پنجه هستند و هر كدام در ساختار دروني آن از مواد و مصالح مخصوص خود استفاده مي نمايند. چهارده نفر از افراد استفاده كننده از اين نوع پنجه به صورت تصادفي انتخاب شدند كه همگي مرد بوده، قطع عضو تروماتيك داشتند. مواد و روش‌ها: نوع مطالعه شبه تجربي و با اندازه گيري مكرر است. اين مطالعه بر روي چهارده نفر از افراد مبتلا به قطع عضو زير زانو انجام گرديد. دامنه سني 25 تا 50 سال و همگي مرد بودند. پرسشنامه اي از نوع بسته طراحي شد و اطلاعات مربوط به هر شخص در پرسشنامه وارد گرديد. در مجموع هر فرد دو جلسه مراجعه نمود: دو هفته پس از نصب پنجه نوع اول (ايراني) و دو هفته پس از نصب پنجه نوع دوم (آلماني). افراد در مقابل دستگاه آناليز راه رفتن عبوركرده، پس از ثبت نتايج در هر مرحله توسط دينامومتر نيروهاي وارده اندازه گيري و ثبت گرديد. با تكميل پرسشنامه‌ها مقايسه يافته‌ها و سپس آناليز داده ها در نرم افزار SPSS انجام پذيرفت. نتايج: مقايسه ميانگين سرعت حركت، شتاب حركت و نيروي تجمع يافته بر روي پنجه در بين  14 نفر هنگام استفاده از دو پنجه ايراني و آلماني  انجام شد و مشخص گرديد سرعت مفاصل هيپ، زانو و مچ پا در تمامي موارد در پنجه آلماني به صورت معني­داري بيشتر از نوع پنجه ايراني است. شتاب درمفصل هيپ در ثانيه 2 و 1 در پنجه آلماني از ايراني بالاتر است اما در ثانيه سوم اختلاف معنادار نيست. شتاب مفصل زانو تنها در ثانيه دوم حركتي معني­دار بوده و به نفع پنجه آلماني است. توزيع نيرو در قسمت قدامي پنجه در نوع آلماني به صورت معني­داري از نوع ايراني كمتر است. بحث: در مقايسه، خصوصيات پنجه آلماني شامل شتاب، سرعت و نيرو در مفاصل مختلف نسبت به پنجه ايراني به صورت نسبي بيشتر است.

Title: تهیه و تعیین پایایی و روایی فهرستی از واژگان زبان فارسی برای دستگاه SNORS

http://jrrs.mui.ac.ir/index.php/jrrs/article/view/98

چکیده مقدمه: یکی از شایعترین مشکلات در بیماران مبتلا به اختلال تشدید خروج هوا از بینی است. Super Nasal Oral Ratio System (SNORS) وسیله­ای است که نسبت خروج هوا از دهان و بینی را تعیین می­کند. با مقایسه نسبت خیشومی بیمار  با داده­های هنجار، می­توان قضاوت دقیقی در مورد بیماران مبتلا به اختلالات تشدید داشت. بدین منظور، باید 10 واژه دارای روایی وپایایی در  زبان فارسی تهیه کرد. مواد و روش­ها: این مطالعه از نوع توصیفی- تحلیلی بود.  بعد از طی مراحل مختلف، 30 واژه انتخاب شد. روایی محتوایی این واژه­ها توسط آسیب شناسان گفتار و زبان تأیید شدند. از37 آزمودنی(22 زن، 15 مرد) خواسته شد که واژه ها را 10 بار بخوانند. نسبت خیشومی هر واژه توسط دستگاه SNORS تعیین می­شد. به منظور تعیین پایایی واژه­ها، دو هفته بعد آزمون دوباره تکرار شد. نتایج: اختلاف معني­داری در نسبت خیشومی هر یک از واژه ها بین دو جنس وجود نداشت. بررسی نتایج آزمون و تکرار آن نشان داد که اختلاف معني­داری بین نسبت خیشومی 22 واژه وجود نداشت. اما در 8 واژه دیگر، اختلاف معني­داری مشاهده شد. از بین این 22 واژه، 10 واژه (5 واژه دارای همخوان خیشومی و 5 واژه بدون همخوان خیشومی) که دارای کمترین انحراف معیار بودند، به عنوان فهرست نهایی برگزیده شدند. بحث: فهرست نهایی را می­توان به عنوان مقیاسی برای تعیین نسبت خیشومی در دو جنس، با استفاده از SNORS به کار برد. پایایی آزمون با تحلیل آماری و روایی آن توسط اساتید گروه آسیب شناسی گفتار و زبان تأیید شد.

Title: بررسي تأثير ارتزهاي رايج بر میزان درد افراد مبتلا به ضايعه آرنج تنيس بازان

http://jrrs.mui.ac.ir/index.php/jrrs/article/view/99

چکیده مقدمه: در مبتلایان به ضایعه آرنج تنیس بازان، به دلیل وجود درد در ناحيه خارجي ساعد بسیاری از حرکات روزمره مختل مي‌گردد. يكي از روشهاي درماني رايج جهت اين عارضه استفاده از ارتز است. هدف عمده استفاده از ارتزهای نيروي متقابل، فراهم نمودن عملکرد فوري و كاهش درد آني است. چون با استفاده از اين ارتزها درد کاهش یافته، عملکرد دست افزایش می­یابد. مواد و روش‌ها: مطالعه حاضر شبه تجربی از نوع كارآزمايي باليني است. مراجعه كنندگان 50 فرد (30 زن و20 مرد با ميانگين سني 8±41 سال) مبتلا به ضايعه آرنج تنيس بازان شهر اصفهان بودند. میزان درد از طريق آزمون سنجش درد (VAS) جهت هر بیمار بعد از بستن هر ارتز اندازه‌گيري و ثبت شد. ارتزهاي مورد استفاده در اين مطالعه شامل تسمه دورآرنج، آستين واره، مچ‌بند و پلاسبو (پاتلای استرپ تغيير شكل يافته) بود. اطلاعات مربوط به میزان درد، با استفاده از نرم افزار SPSS  نسخه یازدهم، مورد تحليل آماري قرار گرفت. آزمونهاي آماري مورد استفاده در اين مطالعه شامل آزمونهاي t مستقل و زوج بود. نتایج: ميانگين سني افراد 8±41 سال بود مقایسه مچ بند طبي با تسمه دور آرنج، همچنين مچ بند با آستین واره نشان می‌دهد که تفاوت معني­داری بین میانگین دو گروه وجود دارد (05/0 > P). نتايج كلي نشان مي‌دهد كه تسمه دور آرنج و آستین واره هر دو باعث کاهش درد شده­اند (05/0 < P). بحث: عملكرد تسمه دور آرنج و آستين واره در حين ارزيابي میزان درد به نسبت مشابه با يكديگر بود و بهترين تأثير را  بر روي ضايعه داشتند. مچ بند طبي نيز در كاهش درد مبتلايان مؤثر بوده ولي تأثير آن كمتر از تسمه دور آرنج و آستين واره بوده است.

Title: تعیین و مقایسه میانگین و دامنه تغییرات بسامد پایه، آشفتگی بسامد پایه و آشفتگی شدت در صوت مبتلایان به فلج یک طرفه تارآواها و اشخاص عادی

http://jrrs.mui.ac.ir/index.php/jrrs/article/view/100

چکیده مقدمه: فلج تارآواها از آسیب­های به نسبت شایع در ایجاد اختلال صوت است. اما درباره این که ابعاد مختلف صوت چقدر تحت تأثیر این اختلال قرار می‌گیرد، اطلاعات زیادی در دست نیست. در این ‌باره به نظر می­رسد اندازه‌گیری آزمایشگاهی، ابعاد مختلف صوت، نقش این بیماری را در اختلال صوت مشخص‌تر می­کند. در این مطالعه با توجه به این که اندازه‌های معتبر و قابل قبول همگان از ابعاد مختلف صوت طبیعی در جامعه در دست نیست، صدای بیماران مبتلا به فلج یک طرفه تارآواها با افراد عادی مورد مقایسه قرار گرفته است. مواد و روش‌ها: در یک مطالعه مقطعی و توصیفی ـ تحلیلی، 10 بیمار مرد مبتلا به فلج تارآواها که به طور غیر تصادفی و از میان افراد مراجعه کننده به بیمارستان حضرت رسول (ص) تهران انتخاب شدند، مورد مطالعه قرار گرفتند و برخی ویژگی‌های اکوستیکی صوت در آنها با گروه شاهدشان مقایسه شد. نمونه صوت آزمودنی‌ها با استفاده از نرم افزار Dr Speech مورد بررسی قرار گرفت و ویژگی‌های اکوستیکی مورد نظر در آنها تعیین شد. سپس از آزمون t مستقل به منظور مقایسه بین دو گروه استفاده شد. نتایج: میانگین بسامد پایه در خواندن جملات و دامنه تغییرات آن  در دو گروه  با یکدیگر تفاوت معنی­داري نداشت. اما میانگین آشفتگی بسامد پایه و میانگین آشفتگی دامنه شدت در واکه /a/  در دو گروه با یکدیگر تفاوت معنی­داري داشت. بحث: آسیب به تارآواها موجب نامتقارن شدن آنها می‌شود که این نامتقارن بودن خود موجب می‌شود تا دو تارآوا با دو سرعت متفاوت به ارتعاش درآیند و دو بسامد پایه متفاوت ایجاد شود. بنابراین پارازیت‌هایی در صدا ظاهر می‌شود. این پارازیت‌ها در آزمودنی‌های مورد مطالعه در بسامد‌های بالا ظاهر شده‌اند و در حقیقت بیشینه بسامد پایه بالا بوده و به همین دلیل میانگین بسامد پایه آنها بالا رفته است. بیشتر بودن دامنه تغییرات بسامد پایه به علت تفاوت ساز و کارهای حنجره‌ای است. آشفتگی بسامد ناشی از تغییرات  لحظه­ای، غیر ارادی و تصادفی در دستگاه آواسازی است که در حنجره آسیب دیده و تارآواهای نامتقارن بیشتر روی می‌دهد؛ آشفتگی شدت به عنوان یک تغییر ناگهانی و غیر ارادی در شدت می­باشد و شدت صدا نیز تحت تأثیر فشار هوای زیر چاکنای است. به علت وجود ضایعه در تار صوتی، فشار هوای زیر چاکنای به درستی کنترل نمی­شود و بروز تغییرات ناگهانی در شدت افزایش می­یابد.

Title: بررسي مقايسه اي تاثير تمرين درماني بر روي عملكرد راه رفتن و بالانس در افراد همي پلژي سمت راست و سمت چپ

http://jrrs.mui.ac.ir/index.php/jrrs/article/view/101

چکیده مقدمه: به اختلال در عملكرد سيستم عصبي كه به دنبال اختلال در خون رساني مغز ايجاد شده سكته مغزي مي‌گويند. سكته مغزي مهمترين عامل در ايجاد ناتواني و معلوليت در بالغين مي باشد. يكي از بزرگترين ناتواني‌ها پس از سكته ناتواني در راه رفتن است به طوري كه بيش از50% از بيماراني كه مرحله حاد بيماري را طي مي­كنند در راه رفتن ناتوان مي­باشند و به يك دوره توانبخشي براي رسيدن به يك سطح عملكردي براي راه رفتن نياز دارند. مواد و روشها: به منظور مقايسه تأثير تمرين درماني بر روي عملكرد راه رفتن، بالانس و بهبود افسردگي در افراد مبتلا به همي پلژي سمت راست و چپ، كارآزمايي باليني غيرتصادفي انجام گرفت. در اين مطالعه از 13 بيمار همي پلژي سمت راست با ميانگين سني (47/1 ± 46/60) و 13 بيمار همي‌پلژي سمت چپ با ميانگين سني (01/2 ±  92/61) كه از نظر سن، قد و وزن همگن شده بودند، استفاده گرديد. به منظور بررسي عملكرد راه رفتن از مقياس Dynamic Gait Index، عملكرد بالانس مقياس Berg balance scale و افسردگي پرسشنامه افسردگي بك استفاده گرديد. جهت تجزيه و تحليل آماري اطلاعات به دست آمده از نرم افزار SPSS استفاده گرديد. به منظور مقايسه تأثير تمرين درماني در بهبود عملكرد راه رفتن و بالانس و بهبود افسردگي قبل و بعد از درمان در هر گروه از آزمون t زوج شده و جهت مقايسه بين دو گروه از آزمون t مستقل استفاده شد. نتايج: با انجام تجزيه و تحليل بر روي داده هاي به دست آمده نتايج زير حاصل گرديد:از نظر عملكرد راه رفتن در گروه همي پلژي سمت راست (001/0 < P) و همي پلژي سمت چپ (001/0 < P) قبل و بعد از درمان تفاوت معني­داري حاصل گرديد.از نظر عملكرد بالانس در گروه همي پلژي سمت راست (01/0 < P) و همي پل‍ژي سمت چپ (001/0 < P) قبل و بعد از درمان تفاوت معني­داري حاصل گرديد.از نظر بهبود افسردگي در گروه همي پلژي سمت راست (001/0 < P) و همي پل‍ژي سمت چپ (001/0 < P) قبل و بعد از درمان تفاوت معني­داري حاصل گرديد.از نظر عملكرد راه رفتن قبل (85/0 = P) و بعد از درمان (35/0 = P)  بين دو گروه تفاوت معني­داري يافت نشد.از نظر عملكرد بالانس قبل (056/0 = P) و بعد از درمان (66/0 = P)  بين دو گروه تفاوت معني­داري يافت نشد.از نظر  بهبود افسردگي قبل (067/0 = P)  و بعد از درمان (054/0 = P)  بين دو گروه تفاوت معني­داري يافت نشد. بحث و نتیجه‌گیری: نتايج حاصل از اين مطالعه نشان داد كه انجام پروتكل تمرين درماني باعث بهبود عملكرد راه رفتن و بالانس و بهبود افسردگي در افراد مبتلا به همي پلژي مي شود اما تفاوتي بين دو گروه همي‌پلژي سمت راست و چپ حاصل نگرديد.

Title: شناخت اجتماعي در كودكان مبتلا به آسيب زباني ويژه

http://jrrs.mui.ac.ir/index.php/jrrs/article/view/102

چکیده مقدمه: شناخت اجتماعي منعكس كننده نحوه ادراك فرد از احساس و تفكر خود و ديگران مي‌باشد. اين سازه شناختي، انعكاسي ـ مشاركتي و داراي كنشهاي سازشي است كه در سه مقوله ادراك اجتماعي، قضاوت اجتماعي و حافظه اجتماعي در حيطه‌هاي مختلف رفتاري انسان مورد مطالعه قرار مي‌گيرد. هدف اين مقاله مرور پژوهش‌هاي مرتبط با اين سازه در مبتلايان به آسيب ويژه زبان است. مواد و روش‌ها: اين مقاله مروري است برمطالعات مرتبط با موضوع از سال 1997 تا 2005 به قصد دستيابي به يافته‌هاي مشترك و نيز سؤالات مطرح شده در اين بررسي‌ها براي تصريح بيشتر دورنماي پژوهشي در اين حيطه. نتايج: مطالعات مختلف بيانگر ارتباط مهارتهاي زباني برتر با كفايت اجتماعي - شناختي است، بر اين مبنا كودكان مبتلا به آسيب‌هاي زباني از جمله آسيب ويژه زبان در دانش شناخت اجتماعي دچار ضعف مي‌باشند. بحث: گو اين كه اكثر مطالعات دانش زبانيِ محدود را تنها عامل ضعف در كفايت اجتماعي نمي‌دانند لكن با تأكيد بر ضعف مبتلايان به SLI در مهارت‌هاي گفتگو، حل تعارض‌هاي بين فردي و برقراري روابط با همسالان، به پديده خود ادراكي منفي و عزت نفس پايين بويژه در عملكرد اجتماعي و عزت نفس تحصيلي اشاره نموده و اهميت مطالعه بيشتر در اين حيطه را ضروري دانسته‌اند.

Title: بررسي تعادل و پاسچر در بيماران مبتلا به كمر درد

http://jrrs.mui.ac.ir/index.php/jrrs/article/view/103

چكيده کمر درد يکی از مهمترين اختلالات سيستم عصبي ـ عضلاني است که به خصوص در جوامع صنعتی مشاهده مي­شود. به همين دليل مطالعات فراوانی در مورد عامل بروز و راههای پيشگيری و درمان آن انجام شده است. به دليل آن كه تعادل مناسب جهت انجام فعاليتهاي روزمره (ADL) لازم و ضروري است، ارزيابي تعادل در بيماران مبتلا به كمردرد بسيار مهم مي باشد. اين بيماران دچار نقص در اجزاي فيزيولوژي آوران و وابران كنترل كننده تعادل و كاهش سفتی، قدرت و استقامت عضلات هستند. اختلال در تعادل در بيماران مبتلا به كمردرد به طور مكرر گزارش شده است. حفظ تعادل نيازمند استراتژيهاي حسي، پردازش حركتي و بيومكانيكي است. پيامهای حسي مورد نياز جهت كنترل تعادل و پاسچر شامل سه دست­اند: 1) پيامهای حسي مخابره شده توسط سيستم  بينايي، 2) پيامهای حسي مخابره شده توسط سيستم  وستيبولار، 3) پيامهای حسي مخابره شده توسط گيرنده هاي حس عمقي. حس عمقي يكي از مهمترين قسمت‌ها مي­باشد زيرا شامل حس تشخيص وضعيت مفاصل، حس تشخيص حركت  مفاصل، حس تشخيص نيرو، سنگيني مربوط به انقباض عضله و زمان انقباض عضله است كه اين اطلاعات از ناحيه دوك عضلاني و گيرنده‌هاي موجود در مفاصل و پوست به سيستم عصبي مركزي مخابره مي­شود.طبق نتايج به­ دست آمده از تحقيقات اختلال در حفظ تعادل و كنترل پاسچر، اختلال در توانايي دوباره‌سازي وضعيت و اختلال در احساس حركت در بيماران مبتلا به كمردرد مزمن را مي‌توان ناشی از تغيير در اطلاعات مخابره شده توسط گيرنده هاي مكانيكي، اختلال در عملكرد دوكهاي عضلات پارا اسپينال، اختلال در قدرت و هماهنگي انقباضات عضلاني، تأخير در فعال‌سازي عضلات تنه و يا  افزايش تنشن فعال عضلات در اين بيماران دانست که در بين عوامل ذکر شده تغيير در حس عمقی را مي­توان يکی از موارد مهم در بروز اختلال در تعادل در بيماران مبتلا به کمردرد به شمار آورد

Title: گزارش موردي يك كودك مبتلا به ديسفاژي دهاني با منشأ سرخجه مادرزادي

http://jrrs.mui.ac.ir/index.php/jrrs/article/view/106

چکیده مقدمه: بيمار دختري است كه حاصل حاملگي فول ترم و روش سزارين بوده است. از ابتدا مشكلاتي در تغذيه و بلع داشت. ميكروسفالي او قابل توجه بود و به موازات آن تاخير در رشد  و نمو (growth, development)   نيز در همه مراحل رشد ديده مي‌شد. در  بررسي‌هاي انجام شده سي‌تي‌اسكن طبيعي و آزمايشات مويد سندرم سرخجة مادرزادي بوده است.در سن 5 سالگي به­ دليل مشكلات زيادي در تغذيه و كمبود وزن به كلينيك گفتار درماني ارجاع داده شد. در ارزيابي feeding  مشخص شد كه عملكردهايchewing  وbiting را دارا نيست و بلع او نيز غير طبيعي است. رژيم غذايي او به چند مادة خاص غذايي محدود بود كه تنها از طريق مكيدن انجام مي‌شد. طول مدت تغذيه در هر وعده غذايي حدود يك ساعت بود كه در اين مدت به دفعات دچار آسپيراسيون و حالت تهوع مي‌شد.زير‌سيستمهاي گفتاري شامل تنفس، آواسازي، توليد و تشديد و در نتيجه گفتار او طبيعي (متناسب با سن و جنس) بود. به تشخيص آسيب‌شناس گفتار و زبان، مشكل اصلي او ديسفاژي دهاني بود. بنابراين برنامه درماني او كه شامل تمرينات دهاني ـ حركتي، درمانهاي مايوفانكشنال، آموزش والدين و روشهاي اصلاح رفتار  بود به مدت سه ماه، دو بار در هفته اجرا شد. نتايج: پس از سه ماه برنامه درماني منظم و همكاري خانواده، مهارتهاي biting و chewing ايجاد شده، جايگزين مكيدن شد. آسپيراسيون و حالت تهوع در طول تغذيه از بين رفت. طول مدت تغذيه از يك ساعت به نيم ساعت كاهش و تمايل او به خوردن مواد غذايي متنوع افزايش يافت. بحث: نتايج اين بررسي نشان داد كه تركيبي از تمرينات دهاني ـ حركتي، درمانهاي مايوفانكشنال، آموزش والدين و روشهاي اصلاح رفتار مي‌تواند در درمان ديسفاژي دهاني مفيد و مؤثر باشد.
